# Chronic Testicular Pain and Foreign Object in Penis: Cultural and Clinical Insights on Artificial Penile Implants

**DOI:** 10.1007/s11606-024-09344-y

**Published:** 2025-01-13

**Authors:** Lauren Elizabeth Damon, Mithu Molla

**Affiliations:** Davis Medical Center, Division of Hospital Medicine, University of California, 2315 Stockton Blvd, Ste 2P101, Sacramento, CA 95817 USA

A 55-year-old man presented with several months of progressive testicular pain. Physical exam was notable for a freely mobile, firm subcutaneous nodule in the shaft of the penis tender to palpation (Fig. 1). CT of the abdomen and pelvis demonstrated a subcutaneous foreign object in his penis (Fig. 2). Scrotal ultrasound was unremarkable. Chlamydia and gonorrhea were negative. He reported that the object was a bead inserted 20 years prior to please sexual partners. Urologic consultation and bead explantation were offered but declined.

**Figure 1:**
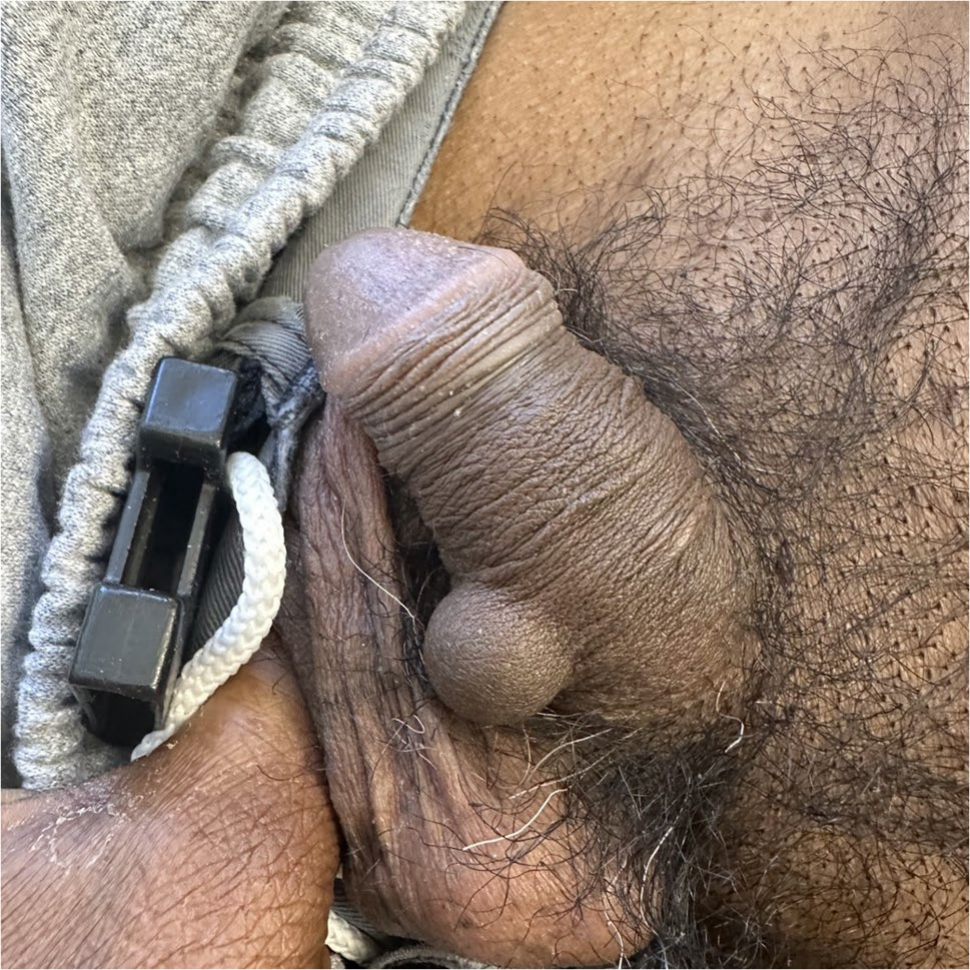
Photograph of the artificially implanted penile bead.

**Figure 2:**
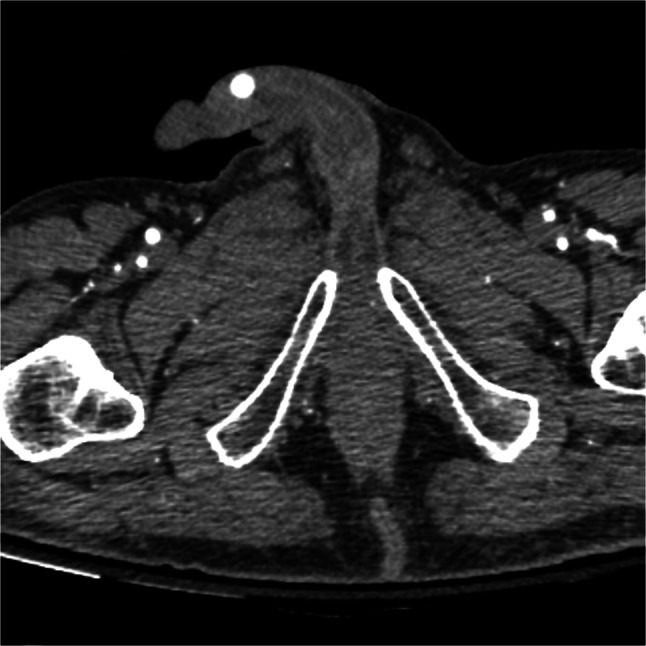
CT abdomen/pelvis demonstrating bead implanted into the subcutaneous tissue of the penis.

Implantation of artificial penile beads is a cultural practice seen most in Slavic and Asian cultures. Prevalence of implants varies widely from 0.5 to 22% depending on the population studied.^1^ Reasons for implantation include establishing status within a subculture, pleasing sexual partners, and rarely causing harm to sexual partners.^1^ A majority of implantations are uncomplicated, but potential complications include penile pain,^2^ delayed secondary infections,^2^ wounds, condom breakage,^3^ and higher risk of sexually transmitted infections (STI).^3^ Complications may impact sexual partners, including wounds, bleeding, dyspareunia, and increased risk of STIs.^3^ Physicians of all backgrounds need to be aware of potential complications associated with penile implantations and culture significance of this practice to approach patients with cultural humility and respect.

## References

[1] **Fischer N, Hauser S, Brede O, et al.** Implantation of Artificial Penile Nodules—A Review of Literature. J Sex Med. 2010; 7:3565–3571. 20102449 10.1111/j.1743-6109.2009.01659.x

[2] **Marzouk D.** Implantation of Beads in the Penile Skin and its Complications. Scand J Urol Nephrol. 1990; 24:167-169 2237292 10.3109/00365599009180852

[3] **Thomson N, Sutcliffe CG, Sirirojn B, et al.** Penile modification in young Thai men: risk environments, procedures and widespread implications for HIV and sexually transmitted infections. Sexually Transmitted Infections. 2008; 84:195-197. 18192295 10.1136/sti.2007.028530

